# High aspect ratio micro-explosions in the bulk of sapphire generated by femtosecond Bessel beams

**DOI:** 10.1038/srep34286

**Published:** 2016-09-27

**Authors:** L. Rapp, R. Meyer, R. Giust, L. Furfaro, M. Jacquot, P. A. Lacourt, J. M. Dudley, F. Courvoisier

**Affiliations:** 1 Institut FEMTO-ST, UMR 6174 CNRS Université Bourgogne Franche-Comté, 25030 Besancon Cedex, France

## Abstract

Femtosecond pulses provide an extreme degree of confinement of light matter-interactions in high-bandgap materials because of the nonlinear nature of ionization. It was recognized very early on that a highly focused single pulse of only nanojoule energy could generate spherical voids in fused silica and sapphire crystal as the nanometric scale plasma generated has energy sufficient to compress the material around it and to generate new material phases. But the volumes of the nanometric void and of the compressed material are extremely small. Here we use single femtosecond pulses shaped into high-angle Bessel beams at microjoule energy, allowing for the creation of very high 100:1 aspect ratio voids in sapphire crystal, which is one of the hardest materials, twice as dense as glass. The void volume is 2 orders of magnitude higher than those created with Gaussian beams. Femtosecond and picosecond illumination regimes yield qualitatively different damage morphologies. These results open novel perspectives for laser processing and new materials synthesis by laser-induced compression.

Under extreme focusing in wide bandgap materials such as glass or sapphire crystal, femtosecond laser pulses create spherical nano-voids^1^,^2^,^3^,^4^. Such structures are of significance not only for 3D structuring applications such as 3D woodpile photonic crystals^5^, but are also of fundamental interest since the pressure created by the plasma expansion is high enough to generate new material phases. This was already reported around the voids produced in sapphire crystal^6^. However, the typical diameter of the void is a few hundreds of nanometers and the amount of material created is extremely limited.

It has been recently demonstrated that Bessel beams can be used to reduce the nonlinear distortions usually suffered by Gaussian beams^7^. In particular, the use of a Bessel beam allows for the intensity clamping effect to be overcome for sufficiently high cone angles, and a Bessel–shaped femtosecond pulse creates a plasma track that is extended and with high free-electron density^8^. The resulting high energy density deposited by the pulse allows the drilling of high aspect ratio nanochannels with single pulses^9^,^10^,^11^. Other groups reported void formation in the bulk of PMMA with Gaussian beam elongated filaments^12^,^13^, This was also reported for fused silica, but only with picosecond illumination^14^. However, it was noticed in our early experiments^9^ that in Corning 0211 glass, the opening of nanochannels was possible only when the Bessel zone, i.e. the field of interference, was not entirely located within the sample, but rather part of the zone crossed the sample interface with the surrounding air, thus allowing material ejection out of the bulk. When focusing the beam entirely within the bulk of the sample, only index modifications were visible^9^. It was unclear if the reason for absence of void inside glass was due to the limited energy density deposited by the femtosecond pulse or because of the closing of the channel by melted material.

Here, we investigate Bessel beam illumination of sapphire, where melting effects are reduced. This material has a thermal conductivity that is ~25 times higher than fused silica and Corning 0211 glass^15^. Therefore, faster cooling is expected with reduced the heat affected zone. We report high aspect ratio nanochannel and void formation in the bulk of the material, with direct observations by Scanning Electron Microscopy (SEM) after Focused Ion Beam (FIB) milling of the laser-processed areas. On the other hand, we report different behavior with picosecond illumination: although high aspect ratio voids can be formed with dimensions close to those obtained by femtosecond illumination, the voids are surrounded by a relatively thick shell of resolidified material, with debris apparent within the void.

## Results

### Experimental procedure

Our experimental setup is described in detail in refs 9 and 16 and in the Methods section. The beam of a 140 fs amplified Ti:Sapphire laser with a central wavelength of 800 nm is shaped into a Bessel beam by a Spatial Light Modulator. This creates a high-quality Bessel beam with cone angle 26°, corresponding to a central spot-size of 0.7 μm FWHM with a Bessel zone extending over ~32 μm in air. The dispersion of the compressor has been adapted to compensate the dispersion in the optics to reach a pulse duration of 140 fs at the sample site. A zero-order half-waveplate and quarter waveplate allow for controlling the beam polarization.

The sample positioning is controlled by a 5 axis stage with an accuracy better that 1 μm^17^. The sample is monocrystalline sapphire (Shinkosha Co), C-cut, double-side polished with a thickness of 100 μm. All experiments described below are obtained with single shot illumination. After laser processing, the structures were cut-through by FIB-milling (FEI Helios 600i). Specific care was taken to avoid any supplementary damage created by FIB milling (see Methods section).

### Presence of voids in the bulk produced by single laser shots

The threshold for femtosecond single shot damage in sapphire was determined by optical microscopy (NA 0.8, x100 microscope objective). The value of the threshold is 1.2 μJ for material index modification and for energies higher than 2.4 μJ, cracks were apparent and typically extended over several micrometers in width. We have therefore chosen a pulse energy of 2 μJ for all experiments described below. The energy density necessary to generate the void with Bessel beams is of the same order of magnitude as reported in refs 4 and 6 for the case of strong focusing of femtosecond Gaussian beams.

**Figure 1:**
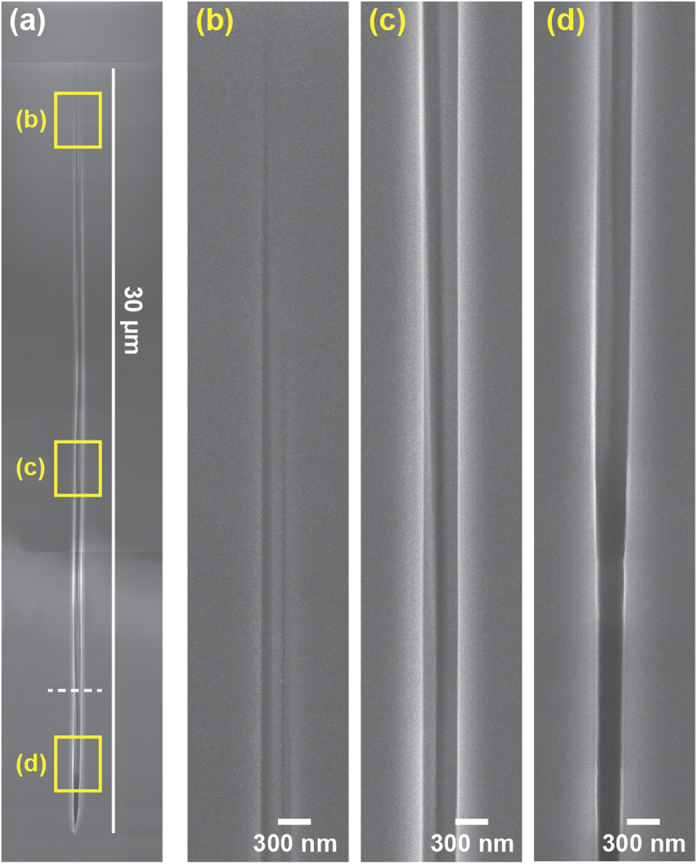
(**a**) SEM imaging of a nano-void inside sapphire created by a single 2 μJ femtosecond pulse. The structure is fully enclosed within the sapphire and has been opened by FIB milling. (**b–d**) Are magnified views of the void for the regions indicate in (**a**). The white dashed line corresponds to the cross section cut shown in Fig. 3 below.

Figure 1(a) shows a high resolution SEM image of a void created in the bulk of sapphire and opened by FIB milling. The void is ~300 nm in diameter and extends over 30 +/− 3 μm, with a progressive reduction of the diameter at the extremities. The large error bar is due to the difficulty of determining the boundaries of the void in the vertical direction. Figure 1(b–d) show magnified views of the inside of the channel: it is very clear that the channel is free from debris with vertical parallel walls. While damages and compression zone around the void are expected it appeared to be difficult to observe them with the SEM. The volume of the void is ~2 femtolitres, which is 2 orders of magnitude larger than the voids created from Gaussian beams (~10 attolitres)^4^.

**Figure 2:**
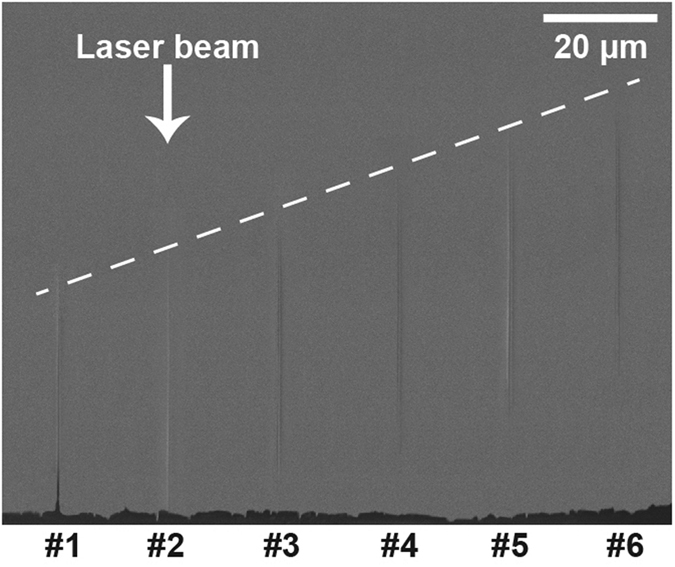
Side view SEM image of the sapphire sample with opened nanochannels using single pulse illumination. The opening was made by FIB milling, and the depth of beam focus was linearly varied by 4 μm between each pulse. The incident laser beam direction is marked as the white arrow, and the depth variation is indicated as the white dashed line. The energy per pulse was 2 μJ at 140 fs and a wavelength of 800 nm.

Figure 2 shows structures produced at varying input positions of the beam with respect to the surface. The spacing between the pulses used was 20 μm. No noticeable difference was observed in the void diameters obtained. However, we observed that the void with an exit hole (numbered #1) is slightly longer than the other ones by ~3 μm. This can be understood because of the smaller energy density necessary to remove material in this configuration compared to bulk focusing. The energy density threshold to evacuate the material is reached over a longer distance than for the bulk focusing.

### Asymmetry of the voids

**Figure 3:**
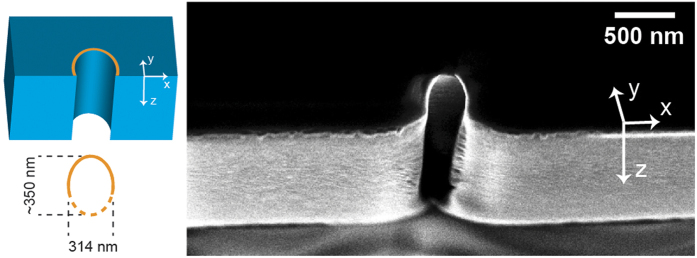
SEM image of the cross section of the void channel of Fig. 1(a) at the position of the white dashed line. The cross section was revealed by transverse FIB milling of the lower part of the void. The laser electric field is polarized along y direction. (left) Schematic reconstructed cross-section profile with measurements.

We carefully examined the cross section of the channels and Fig. 3 shows an image of the transverse section of the void structure shown at the position of the white dashed line in Fig. 1.

As shown in the inset, the cross section of the void is elliptical with a major axis of approximately 350 nm (oriented along y direction, parallel to the polarization of the incident field) and a minor axis of 314 nm. This asymmetry is reported for the first time to our knowledge and does not arise from the optical anisotropy of the crystal because the ellipticity of the channels is also observed on the top surface of the sapphire sample, where optical propagation in the anisoptropic medium is negligible. Rotating the polarization rotates by the same amount the main axis of the elliptical cross-section, while rotating the crystal has no effect on the top surface damage. Further experimental work is currently ongoing to reveal its origin.

### Influence of pulse duration

**Figure 4:**
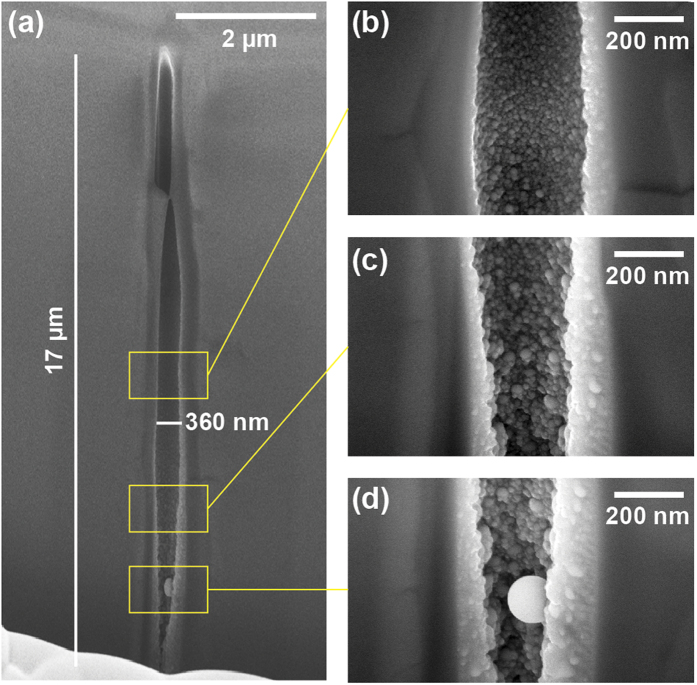
SEM images of an elongated void structure in the bulk of sapphire for picosecond regime illumination. Parameters were 2 μJ pulse energy and of 3 ps duration, and using linear polarisation. (**a)** Shows full structure while (**b–d**) show magnified areas. The void morphology greatly differs from the one of femtosecond regime.

**Figure 5:**
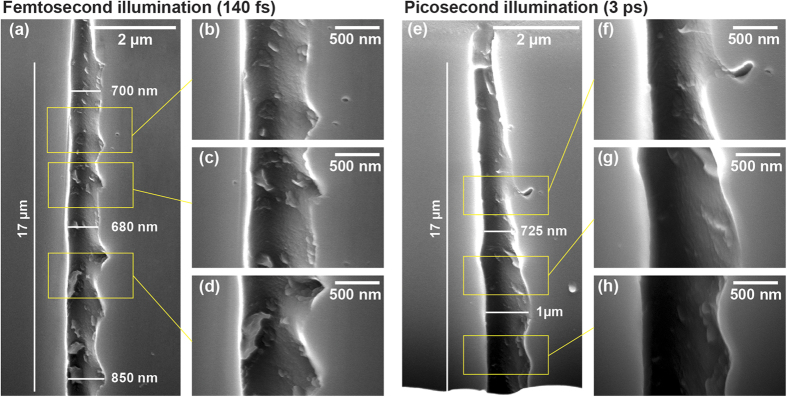
Scanning electron microscope images of the void channels after HF etching. (**a–d**) Femtosecond regime (channel presented in Figs 1 and 3), (**e–h**) picosecond regime (channel presented in Fig. 4).

We repeated the high-aspect ratio microexplosion experiments using longer pulses of 3 ps duration obtained by stretching the pulse in the compressor of our chirped pulse amplification laser source. Under these conditions, the threshold for sapphire index modification was 0.6 μJ and the threshold for crack formation was 2.3 μJ. Even under bulk focusing conditions, it is possible to generate void structures with ps single pulses. To enable comparison with the femtosecond regime, we show in Fig. 4 the void created by a 2 μJ pulse (the same energy as for the structure shown in Fig. 1). The diameter for the picosecond illumination is slightly higher than in the femtosecond regime, i.e. 350 nm. Also in contrast with the femtosecond regime results, the void cross-section remains circular irrespective of the polarization. However, we note that the channel morphology is very different from the case of femtosecond illumination. In particular, the picosecond regime results show sidewalls that are more irregular, and the presence of a shell of resolidified material can be seen surrounding the void. This shell has a diameter of approximately 700 nm and shows a granular structure as is apparent in Fig. 5(b–d). Finally, we note that the bottom of the void shows more particles than the top part of the void. A possible qualitative interpretation for this could be that the vapor phase has been condensed within the void. In the case of picosecond illumination regime, we understand the absence of asymmetry as originating from the much thicker melted shell around the void channel. In this case, ellipticity cannot be maintained in the liquid phase.

### Sapphire densification

To estimate the diameter over which sapphire has been modified, the structures opened by FIB presented in the previous figures were treated by immersion in HydroFluoric acid (HF) (10% in water) for 5 minutes. For these parameters, we confirmed that the etching of the regions of non-illuminated sapphire was negligible. Figure 5 shows SEM images of the previous nanochannels (Figs 3 and 4) where the modified material has been etched. The average diameter of the etched channel is (a) 700 nm for the femtosecond regime compared to (e) 830 nm for the picosecond regime. We note that even the slight modifications appearing as dark defects in SEM images in Fig. 5(a) are now etched. It is very clear that the damages created by femtosecond illumination are much sharper and thinner than those in picosecond. The origin of the asymmetry between right and left sides of the channel for femtosecond regime is still an ongoing question.

From these measurements, we can deduce an estimation of the minimal material densification that has been reached. We approximate the etched diameter (750 to 800 nm) as the diameter of the compressed shell. In reality, the volume that was etched is much larger than the compressed volume because it also includes the region that was thermally modified, so that the densification is probably underestimated. The void volume is ~2.1–2.9 μm^3^ (for a diameter 300–350 nm, length 30 μm), corresponding to a mass of ~8–11 pg. This material mass has been distributed within a shell with a volume of 9 to 13 μm^3^, so that the shell density is between 4.6 to 4.9 g.cm^−3^ with an increase in material density at least larger than 15%. We note that this is in quantitative agreement with the measurements obtained by other groups with Gaussian illumination (14% density increase) and where the compressed region was shown to contain superdense bcc-Al^6^.

## Conclusion

In conclusion, we have demonstrated the generation of high aspect ratio voids in the bulk of sapphire with single femtosecond and picosecond laser pulses with high-angle Bessel beams. The void volumes created are in the range of femtolitres and we expect that longer Bessel beams could create higher aspect ratio voids. The micro-explosions created material densification of ~15% around the voids. While the void opened in the femtosecond regime is free from particles, many particles produced by condensation were observed for 3 ps pulse illumination. We anticipate these results will foster new research for material compression of large volumes and will impact on femtosecond laser materials processing in sapphire.

## Methods

### FIB milling procedure

After laser processing, the structures were cut-through by focused ion beam milling (FEI Helios 600i). Specific care was taken to avoid any supplementary damage created by FIB milling. The sapphire samples were opened in order to obtain a view of the longitudinal cross-section of the nanochannels. This was performed in three steps. First, FIB milling was performed at high current of 9.3 nA up to a distance of 10 microns from the channels location. Then, the current was decreased from 2.5 nA to 80 pA until a distance of less of a micron from the channel. The final approach was made at the low current of 40 pA to obtain a clean and precise section and stops when the whole nanochannel was clearly seen.

## Additional Information

**How to cite this article**: Rapp, L. *et al*. High aspect ratio micro-explosions in the bulk of sapphire generated by femtosecond Bessel beams. *Sci. Rep.* **6**, 34286; doi: 10.1038/srep34286 (2016).
